# Evidence for association between familial bipolar risk and ventral striatal volume

**DOI:** 10.1016/j.jad.2018.02.015

**Published:** 2018-05

**Authors:** T.M. Lancaster

**Affiliations:** a Neuroscience and Mental Health Research Institute, Cardiff University, Cardiff, UK; b Cardiff University Brain Research Imaging Centre (CUBRIC), School of Psychology, Cardiff University, Maindy Road, Cardiff CF244HQ, UK; c MRC Centre for Neuropsychiatric Genetics and Genomics, Institute of Psychological Medicine and Clinical Neurosciences, Cardiff School of Medicine, Cardiff University, Cardiff, UK

**Keywords:** Bipolar, Major depressive disorder, Genetic risk, Striatum, MRI, Freesurfer

## Abstract

**Background:**

Recent genome-wide association studies (GWAS) of striatal volumes and bipolar disorder (BD) indicate these traits are heritable and share common genetic architecture, however little independent work has been conducted to help establish this relationship.

**Methods:**

Subcortical volumes (mm^3^) of young, healthy offspring of BD (N= 32) and major depressive disorder (MDD) patients (N= 158) were compared to larger healthy control sample (N_RANGE_= 925–1052) adjusting for potential confounds, using data from the latest release (S1200) of the Human Connectome Project. Based on recent GWAS findings, it was hypothesised that the accumbens and caudate would be smaller in offspring of BD, but not MDD patients.

**Results:**

After multiple comparison correction, there was a regional and BD specific relationship in the direction expected (Accumbens: F_2,1067_= 6.244, P_FDR-CORRECTED_= 0.014).

**Discussion:**

In line with recent GWAS, there was evidence supporting the hypothesis that reduced striatal volume may be part of the genetic risk for BD, but not MDD.

**Limitations:**

It cannot be concluded whether this association was specific to BD or consistent with a broader psychosis phenotype, due to a small sample size for offspring of schizophrenia patients. Furthermore, one cannot rule out potential shared environmental influences of parental BD.

**Conclusions:**

The common genetic architecture of BD may confer susceptibility via inherited genetic factors that affect striatal volume. Future work should establish how this relationship relates to specific BD symptomology. This work may also help to dissect clinical heterogeneity and improve diagnosis nosology.

## Highlights

• Genome wide association studies suggest bipolar disorder and striatal brain volumes have shared genetic aetiology.
• Little work has explored the impact of familial bipolar risk on subcortical brain structure using large samples.
• Reduced ventral striatum volume in offspring of bipolar patients, but not offspring of major depressive disorder patients.
• Reduced ventral striatum volume may be a neural antecedent that confers risk specifically to bipolar disorder.
• This observation suggests striatal imaging markers may help to dissect clinical heterogeneity in mood disorders.

## 1 Introduction

While there is considerable evidence that affective disorders such as bipolar disorder (BD) and major depressive disorder (MDD) are heritable, comparatively little is known about how genetic risk for these disorders confer susceptibility. In order to establish putative mechanisms of pathophysiology, neuroimaging studies have explored the impact of genetic risk for these disorders by scanning their unaffected relatives/offspring. These studies present mixed evidence that unaffected relative groups show alterations in brain structure (Ladouceur et al., 2008, McDonald et al., 2006, McIntosh et al., 2004, Nery et al., 2015). Furthermore, these studies are unable to determine how putative alterations in brain structure are related to genetic risk.

Genome-wide association studies (GWAS) now suggest liability for affective disorders is partially conferred by thousands of common loci conferring risk *en masse* (Stahl et al., 2017, Wray and Sullivan, 2017). GWAS also suggest that subcortical brain volumes also have a complex polygenic architecture (Hibar et al., 2015, Satizabal et al., 2017). Considerable evidence suggests that subcortical brain volumes are reduced in BD and MDD cases compared to controls (Hibar et al., 2016, Schmaal et al., 2016), however, less is known about the whether these volumetric reductions are linked to the common genetic aetiology of these disorders. These studies are critical in understanding the role of common genetic variation in psychopathology and putative mechanisms of risk.

Preliminary evidence suggests that the observed volumetric subcortical reductions are not due to genetic aetiology that is shared with schizophrenia (SCZ) or MDD (Franke et al., 2016, Reus et al., 2016, Wigmore et al., 2017). However, the genetic relationship between subcortical brain volumes and bipolar risk is less clear. Recent GWAS show a negative genetic correlation between BD and subcortical brain volumes – specifically in the ventral (accumbens) and dorsal (caudate) striatum (Satizabal et al., 2017). This suggests genetic overlap between the common genetic variants that confer risk to bipolar and contribute to individual differences in striatal brain volume.

In the current study, I aim to support the negative genetic association between bipolar genetic risk and striatal brain volumes. Using data from the Human Connectome Project – Young Adult (HC-HCP) cohort, I aim to explore the impact of familial risk for BD/MDD on the subcortical volumes commonly explored in large genetic and case-control imaging studies (Hibar et al., 2015, Hibar et al., 2016). I expect to observe reduced accumbens and caudate volume in BD offspring compared to individuals without a parental history of BD. Furthermore, one may anticipate that putative reductions are specific to BD and not present for individuals with a familial risk to MDD or SCZ. I suggest that the familial alterations in striatal volumes linked to BD may represent a diagnosis-specific, neural antecedent for BD that may a) be linked common genetic variation and b) may be used to future patient stratification and diagnostic strategies.

## 2 Methods

### 2.1 Participants

Data were drawn from the publicly available repository of the WU-Minn HC-HCP (http://www.humanconnectome.org/), which includes individuals who have parents with MDD, BD or SCZ diagnosis. The scanning protocol was approved by Washington University in the St. Louis's Human Research Protection Office (HRPO), IRB# 201204036. No experimental activity with any involvement of human subjects took place at the author's institutions. Participants were drawn from the March 2017 public data release from the Human Connectome Project (N= 1206). All participants were aged from 22 to 35, for all inclusion/exclusion criteria, see Van Essen et al. (Van Essen et al., 2013). Briefly, the study excluded individuals with a personal history of psychiatric disorder, substance abuse, neurological or cardiovascular disease and associated hospitalization or long–term (> 12 months) pharmacological/behavioural treatment. For a full brief of inclusion/exclusion criteria, please see Supplemental Table 1 in (Van Essen et al., 2013). Participants were excluded from the current analyses if they lacked good-quality structural magnetic resonance imaging data, or had missing relevant interview/questionnaire data. Further information about the HCP pedigree/kinship structure can be found at http://www.humanconnectome.org/storage/app/media/documentation/s1200/HCP_S1200_Release_Reference_Manual.pdf. Individuals were excluded who had at least one parent with a diagnosis of schizophrenia (N= 7). Sample sizes for each group were: HC (F= 534/M= 471); MDD (F= 95/M= 57); BD (F= 22/M= 15), and gender was not overrepresented in any group (χ^2^= 5.046, P = 0.08). The BD and MDD offspring groups were comparable in age, handedness and education (P > 1, in all cases; Table 1). To control for potential confounding, these variables were also all added as covariates into all models.

**Table 1:** Mean ± SD (1 standard deviation) for demographic and subcortical volumes (mm^3^), across the three groups.

	Unadjusted Mean±SD						Adjusted group effects		
	HC Parents		MDD Parent		BD Parent		F_¥_	P	*q*FDR
	Mean	±SD	Mean	±SD	Mean	±SD			
Age	28.78	3.69	29.18	3.68	29.35	3.82	0.675	0.509	N/A
Educ	14.92	1.78	14.76	1.91	14.19	1.98	1.963	0.141	N/A
Hand	66.17	45.07	60.76	45.41	69.73	43.28	1.462	0.232	N/A
Accum	586.63	89.25	561.60	85.16	540.79	77.78	6.245	0.002	0.014
Amyg	1601.74	199.13	1564.29	197.86	1572.56	219.59	0.421	0.656	0.656
Caud	3882.16	479.90	3825.78	460.78	3716.45	392.51	1.326	0.266	0.444
Hippo	4460.57	451.57	4426.48	462.62	4443.58	411.17	0.853	0.427	0.498
Pallid	1435.89	205.17	1391.80	180.03	1388.14	205.02	1.151	0.317	0.444
Putam	5575.09	655.94	5446.84	633.22	5331.21	715.39	1.280	0.279	0.444
Thal	7957.97	815.52	7793.94	804.93	7626.15	742.34	3.169	0.042	0.148

BD (offspring of bipolar patients), MDD (offspring of major depressive patients); HC (offspring of healthy controls with no psychiatric diagnosis). Adjusted group effects: reflect the linear-mixed effect model regressions results. ^¥^ Degree of freedom estimated with Satterthwaite approximation and varying according to regression (denominator DOF range = 1055–1127). *q*FDR; reflects P values, adjusted for False Discovery Rate. Age=Age_in_Yrs; Educ=SSAGA_Education; Hand; Handedness (assessed with the Edinburgh Handedness Scale).

### 2.2 Data acquisition, preprocessing and quality control

Human Connectome Project sample: Images were acquired using a customized Siemens Skyra 3-T scanner with a 32-channel head coil. For details on data acquisition and preprocessing, see Glasser et al. (2013). Subcortical and intracranial volume (mm^3^) were estimated with Freesurfer v5.2 (Fischl, 2012), which were subsequently used for the HC-HCP minimal processing pipeline (Glasser et al., 2013). Seven subcortical volumes previously explored in genomics/psychopathology were averaged across hemisphere and adjusted for intracranial volume (ICV), a method previously established by ENIGMA (Franke et al., 2016, Hibar et al., 2015, Hibar et al., 2016, Schmaal et al., 2016). Outliers were then removed from each bilateral subcortical region of interest using the IQR outlier labelling rule (1.5 × interquartile range (Q3-Q1)) as previously described (Hoaglin and Iglewicz, 1987). Out of the total sample (N= 1206), outlier labelling removed approximately 10% of subcortical volumes (N_RANGE_= 1083–1092; varying per volume).

### 2.3 Statistical inferences

Linear mixed-effects models were estimated in R (https://www.r-project.org/) using the *lmerTest* package, as previously recommended (Carlin et al., 2005, Kuznetsova et al., 2015). Familial risk (HC/MDD/BD) was entered into the model as fixed effect (where 0 =no parent with disorder, 1=at least one parent with the disorder) with age, gender, education level and handedness as potential confounds. To account for kinship, family structure (Family ID) and zygosity (monozygotic twins, dizygotic and unrelated individuals; coded as a percent DNA shared; 1, 0.5, 0, respectively) were entered into each model as random effects, which under the model assumptions, could be freely correlated with each other (Carlin et al., 2005). Independence between these random slopes was assumed, in order to control for potential genetic (as assayed by the random effect of zygosity) and familial environmental (as measured by kinship) correlations. These random effects were modelled to control for potential genetic influence over the phenotypic relationship between familial risk and subcortical brain volumes. P-values were adjusted using the False Discovery Rate (Benjamini and Hochberg, 1995).

## 3 Results

**Fig. 1:**
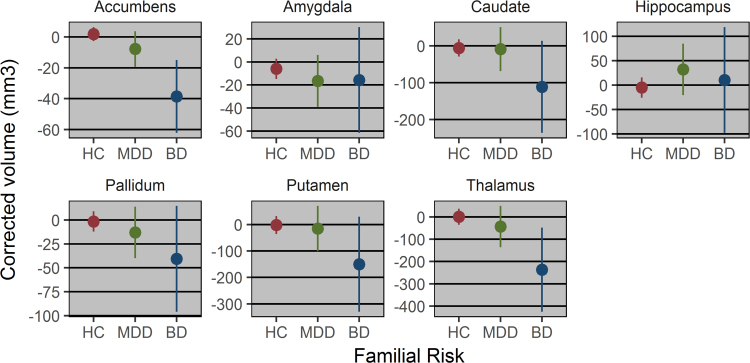
Corrected subcortical volumes (mm^3^) are adjusted for fixed effects (intracranial volume, age, gender, education & handedness) and random effects (kinship, zygosity). Error bars represent 95% confidence intervals. HC=individuals with parents without psychiatric diagnosis; MDD=offspring of major depressive disorder patients; BD=offspring of bipolar disorder patients.

There was a significant association between familial risk and reduced volume (mm^3^) in the nucleus accumbens (F_2,1067_= 6.244, P_FDR-CORRECTED_= 0.014) and uncorrected association in the thalamus (F_2,1055_= 3.169, P_UNCORRECTED_= 0.042). Post-hoc analysis suggested that the group –wise effects on the accumbens were driven by BD offspring (t_911_= −3.171, P = 0.0015) but not MDD offspring (t_1032_= −1.054, P = 0.132), with similar, disorder-specific effects within the thalamus (BD: t_884_= −2.559, P = 0.011; MDD: t_1017_= −0.849, P = 0.396). No other subcortical brain volumes were associated with a familial risk for BD or MDD (P > 0.1, in all cases). See Table 1/Fig. 1 for all estimated effects and 95% confidence intervals.

## 4 Discussion

At present, the relationship between subcortical brain volume alterations and familial bipolar liability is mixed (Nery et al., 2015, Nery et al., 2013). However, our observations are do support early work suggesting that bipolar relatives have reduced thalamic and accumbens volume compared to healthy controls (McDonald et al., 2004, McIntosh et al., 2004). Consistent with a recent observation documented a shared genetic architecture between accumbens/caudate volume and BD (Satizabal et al., 2017), there was a negative association where healthy offspring of BD patients had reduced striatal volume (specifically, within the accumbens) when compared to a large sample of demographically comparable individuals. One could argue that as this observation conforms to the large genetic correlation study (Satizabal et al., 2017), the impact of bipolar risk on accumbens volume may be partially explained by common genetic variation. This hypothesis is also supported by a recent bivariate correlation study showing a genetic relationship between bipolar disorder and accumbens, thalamus and putamen (Bootsman et al., 2015). The role of familial psychiatric risk on subcortical volumes will be further established in large collaborative relative studies such as ENIGMA (Hibar et al., 2016) which will help to identify neural mechanisms by which (genetic) risk increases susceptibility. These studies may be able to establish common and distinct neural antecedents that confer susceptibility across a broad spectrum of psychopathology. Whereas the morphology of the ventral striatum been implicated in the pathophysiology of bipolar disorder, the mechanisms that underpin this association remain relatively unknown. Preliminary evidence suggest that reduced accumbens volume may be linked to comorbid features of bipolar disorder such as stressful, independent life events (Geller et al., 2009), emotion-based impulsivity (Muhlert and Lawrence, 2015) and suicidality (Gifuni et al., 2016).

## 5 Limitations

While familial SCZ was recorded as part of the HCP, there were not enough individuals to include in our analysis (N= 7). As BD and SCZ have considerable genetic overlap, at present it cannot be stated whether the association between parental BD and reduced striatal volumes is unique or a risk feature associated with a broader psychosis phenotype. However, including a fixed term for parental SCZ did not significantly affect any of our observations, and SCZ was not associated with subcortical volumes after adjusting for multiple comparisons. Together with observations that document a lack of genetic overlap between SCZ and brain volumes (Franke et al., 2016), striatal reductions may be a unique feature of bipolar disorder. However, caution is advised when interpreting these observations considering the relatively small BD offspring sample size, compared to the HC and MDD groups. A recent study found no association between polygenic risk for bipolar disorder and striatal volumes, however as risk profile scores (RPS) only explain a small proportion of variance in related phenotypes (Dudbridge, 2013), this study may have been underpowered to detect an effect of polygenic risk. Lastly, due to the design of the present study, one cannot rule out the possibility that shared environmental influence may influence striatal volumes in offspring of bipolar patients. One can also acknowledge that comparative MDD and BD diagnosis groups would have been useful in making comparisons between genetic risk and disease states. However, the study does benefit from limited confounding such as medication, co-morbidity and years with illness, which may influence subcortical volume.

## 6 Conclusion

Our observations suggest that the reductions in ventral striatal volumes observed in patients with BD may be due to genetic factors that increase susceptibility. Our results complement ongoing genomic studies showing negative genetic correlations between striatal volume and BD, but not SCZ and MDD. The volumetric reductions in accumbens volume may be risk factor linked to bipolar specific dimensions of psychopathology and may guide future patient stratification and nosology.
